# Effect of explant source, perlite nanoparticles and TiO_2_/perlite nanocomposites on phytochemical composition of metabolites in callus cultures of *Hypericum perforatum*

**DOI:** 10.1038/s41598-019-49504-3

**Published:** 2019-09-10

**Authors:** R. Ebadollahi, S. Jafarirad, M. Kosari-Nasab, S. Mahjouri

**Affiliations:** 1 Research institute for Fundamental sciences (RIFS), University of Tabriz, Tabriz, Iran; 2 Drug Applied Research Center, Tabriz University of Medical Sciences, Tabriz, Iran; 3 Department of Plant Biology, Faculty of Natural Sciences, University of Tabriz, Tabriz, Iran

**Keywords:** Biotechnology, Plant sciences

## Abstract

It appears that the biologically-synthesized nanoparticles (NPs) have potential to perform as effective elicitors for the production of valuable secondary metabolites in plants. Besides, it has been reported that the toxicity of the biologically-synthesized NP is not as much as that of the chemically-synthesized NPs. Therefore, it is necessary to test their advantages aspects. In this study, the physical synthesis of perlite NPs and biologically-synthesis of TiO_2_/perlite nanocomposites (NCs) were conducted. Subsequently, their effects and explant source influence on the growth characteristics and secondary metabolite profiles of *Hypericum perforatum* callus cultures were evaluated. According to the obtained results, morphology of the synthesized perlite NPs and TiO_2_/perlite NCs were mesoporous and spherical with sizes ranging about 14.51–23.34 and 15.50–24.61 nm, respectively. Addition of perlite NPs and TiO_2_/perlite NCs to the culture medium at the concentration range of 25–200 mg/L showed no adverse impacts on the growth characteristics of *H. perforatum* calli. According to the GC-MS analysis, the stress caused by perlite NPs and TiO_2_/perlite NCs led to an increase in the variety, amount and number of volatile compounds. The calli obtained from *in vitro* grown plants produced more volatile compounds relative to the calli obtained from field grown plants under the nanomaterial stress conditions. The production of hypericin and pseudohypericin were also determined in the callus cultures under desired nanomaterials elicitation. Accordingly, our results suggest that perlite NPs and TiO_2_/perlite NCs can possibly be considered as effective elicitors for the production of volatile compounds, hypericin, and pseudohypericin in callus cultures of *H. perforatum*.

## Introduction

*Hypericum perforatum* L., also known as St. John’s wort, is an important medicinal plant with diverse bioactive constituents such as naphtodianthrones, acyl-phloroglucinols, flavonoids, and xanthones, which have been reported to have anti-inflammatory, antimicrobial, antitumoral, antidepressant and wound-healing activities^1,2^. Production of secondary metabolites by *in vitro* cultures of *H. perforatum* has been one of the most expansively investigated areas^3,4^. However, application of these plant cultures is still limited because of the low yield of the desired compounds. It has been reported that elicitation can be an attractive approach employed to improve the productivity of *in vitro* plant cultures^5^. Quite a lot of abiotic and biotic elicitors have been applied to explore the accumulation of secondary metabolites in cell and organ cultures of *H. perforatum*^6,7^. Recently, nanoparticles (NPs) have been proposed to be a nutrient source and an elicitor, leading to the overproduction of various secondary metabolites^8^. For instance, Poborilova *et al*.^9^ reported the accumulation of phenolics upon the addition of different concentrations of Al_2_O_3_ NPs to the tobacco cell suspension cultures which reached to the maximum level (211.7 µg/g FW) at the concentration of 100 µg/ml after 96 h of treatment. Similarly, artemisinin content was augmented in the 20-day-old *Artemisia annua* hairy root cultures treated with Ag-SiO_2_ core-shell NPs which was 3.9-fold higher than the control^10^. Multi-walled carbon nanotubes (MWCNTs) induced the production of secondary metabolites in *Satureja khuzestanica* callus cultures. The maximum amounts of the total phenolics and flavonoids were determined in the cultures exposed to 100 µg/ml of MWCNTs which were 1.9 and 2.6 times higher in comparison to the control, respectively. Furthermore, the highest content of rosmarinic acid (4.01 mg/g DW) and caffeic acid (2.78 mg/g DW) were obtained at the treatments of 100 and 250 µg/ml in *S. khuzestanica* callus cultures^11^. In the case of *H. perforatum*, an investigation reported that the cultures supplemented with 100 ppb (per 30 ml culture) of zinc nano-oxide showed increased amounts of hypericin (7.87 µg/g DW) and hyperforin (217.45 µg/g DW) when compared with the control (2.07 and 16.27 µg/g DW, respectively). Also, the augmented amounts of hypericin and hyperforin (11.18 and 195.62 µg/g DW, respectively) were observed in *H. perforatum* cultures treated with 100 ppb of iron nano-oxide^12^.

The mechanism through which NPs modulate secondary metabolism is not exactly understood. Genomic analyses have been revealed that plants can respond to the internalization of nano-sized materials like biotic or abiotic stress factors^13,14^. It has been proposed that like the other stressors, NPs can modulate plant secondary metabolism via inducing different cellular signal transduction pathways. Calcium flux, reactive oxygen species (ROS) burst, and mitogen-activated protein kinase (MAPK) phosphorylation can be the primary actions activated by NPs. Generation of ROS has been reported in most of the studies investigating NPs effects on plants^15^. It has been suggested that NP-induced ROS may act as a signal to induce the plant secondary metabolism^16^. Induction of the typical stress signaling reactions, mediated by cytosolic Ca^2+^ and ROS, has been reported in the model plant *A. thaliana* exposed to nanosilver^17^. Similar to animal and human cells, it is estimated that plants may also utilize oxidative stress signaling by using MAPK cascade modules^14^. Therefore, activation of signaling pathways finally leads to gene expression followed by enzymatic reactions, which consecutively change the production of secondary metabolites. Changes in the activities of some enzymes such as phenylalanine ammonia lyase, peroxides, and polyphenol oxidase have been already reported to be related to the biosynthesis of secondary metabolites^18^.

Perlite is a mineral structure with numerous industrial applications. Because of the exceptional properties such as being an inert, porous, low density and cheap material, perlite has been reported to be a suitable support for the immobilization of different catalysts^19,20^. Meanwhile, the immobilization of TiO_2_ NPs on perlite has been suggested to be an appropriate photocatalyst^21^. However, in spite of the varied scope of the applications of these nanomaterials, there is limited information about their impact on plants. Due to the unique properties, for instance large specific surface area and greater reactivity, these ultrafine particles have been considered favorable for many biological applications. Promising properties of perlite in the culture medium, such as improvement of nutrient uptake and aeration, can be used to improve growth and valuable metabolites production in *in vitro* cultures^22^. So far, TiO_2_ NPs have been revealed to have both beneficial and adverse effects on plants, which depends on the physicochemical properties of NPs and the plant species^23,24^. It has been reported that TiO_2_ NPs significantly improved the shoot/root length, chlorophyll content, and total soluble leaf protein of mung bean plant^25^. On the other hand, the increase in yield was observed after treatment of cowpea with TiO_2_ NPs^26^. The highest essential oil content and yield were observed in *Salvia officials* plants exposed to 200 mg/L of TiO_2_ NPs which were 1.75 and 2.74-folds higher than those of the control plants. Besides, the maximum contents of total phenolic (35.2 mg/g DW.) and flavonoid (21.9 mg/g DW) were determined in *S. officials* plants treated with 200 and 100 mg/L of TiO_2_ NPs, respectively^27^.

Biologically-synthesis of NPs has been considered as an important method to reduce the destructive effects of physico-chemical synthesis methods^28^. Moreover, biologically-synthesized NPs are more stable, more effective, and less toxic than chemically-synthesized NPs^15,29^. The physicochemical characteristics of NPs including size, shape, crystal structure, and elemental composition, as well as their biological behavior, can be affected by the utilization of different synthesis methods and diverse reducing and stabilizing materials. Moreover, the final state of the synthesized NPs can be influenced by the interaction with the surrounding media^30^. In this context, it is necessary to use the combination of diverse techniques for characterizing NPs to realize their full potential. In the present study, a physical approach for the synthesis of perlite NPs and a green method using an aqueous extract of *H. perforatum* for the synthesis of TiO_2_/perlite nanocomposites (NCs) were conducted. The properties of the synthesized nanostructures were analyzed using a combination of characterization techniques. Subsequently, the effects of explant source (explants obtained from the field grown plants and explants obtained from *in vitro* grown plants), perlite NPs and TiO_2_/perlite NCs on the growth and secondary metabolite modulation in *H. perforatum* callus cultures were investigated. To the best of our knowledge, this is the first report on the induction of secondary metabolite production by perlite NPs and biologically-synthesized TiO_2_/perlite NCs in callus culture of *H. perforatum*.

## Materials and Methods

### Perlite NPs preparation

Mineral powder of perlite with the chemical composition of Si, 33.8; Al, 7.2; Na, 3.4; K, 3.5; Fe, 0.6; Ca, 0.6; Mg, 0.2; trace elements, 0.2; O2, 47.5 and H_2_O, 3.0 (w/w %) provided from Mianeh area, Iran. This commercial powder was used as starting materials and was intermixed and ball-milled (ceramic balls, 10 h under Ar) at a ball/mill ratio of 10: 1.

### TiO_2_/perlite NCs synthesis

The aerial part extract of *H. perforatum* was used for synthesis of TiO_2_/perlite NCs. About 10 g of the dried plant material was mixed with 100 ml of deionized water, followed by shaking (150 rpm) for 48 h at 25 °C and sonication for 20 min. Then, the mixture was filtered and centrifuged. In order to synthesize of TiO_2_/perlite NCs, 1 g of perlite NPs and 3.84 ml of titanium isopropoxy solution were mixed with 50 ml of the plant extract under constant stirring for 2 h. Subsequently, the mixture was adjusted to pH 7 with 1 M NaOH and refluxed for about 9 h at 140 °C. After washing with deionized water for several times, the resulting precipitate was subjected to oven drying for 4 h at 80 °C, followed by heating at 400 °C for 4 h.

### Characterization of the synthesized nanomaterial

The characteristics of the synthesized nanomaterials were identified using UV-Vis spectroscopy (Spekol 1500), X-ray diffraction (XRD) (D500, Siemens Diffractometer-Germany), transmission electron microscopy (TEM) (LEO 906), field emission scanning electron microscopy equipped with energy dispersive X-ray spectroscopy (EDX) (MIRA3 FEG-SEM.), dynamic light scattering (DLS) (Nanotrac Wave), and Fourier transform infrared (FT-IR) spectroscopy (TENSOR27–Brucker) techniques. The as-synthesized samples for TEM analysis were prepared as follows. The samples were dispersed in ethanol and the suspensions were treated in ultrasonic bath for 20 min. Then, a drop of the dilute suspension was placed on a carbon-coated grid. Afterward, it was allowed to dry by evaporation at room temperature.

### Plant material and callus cultures

**Figure 1:**
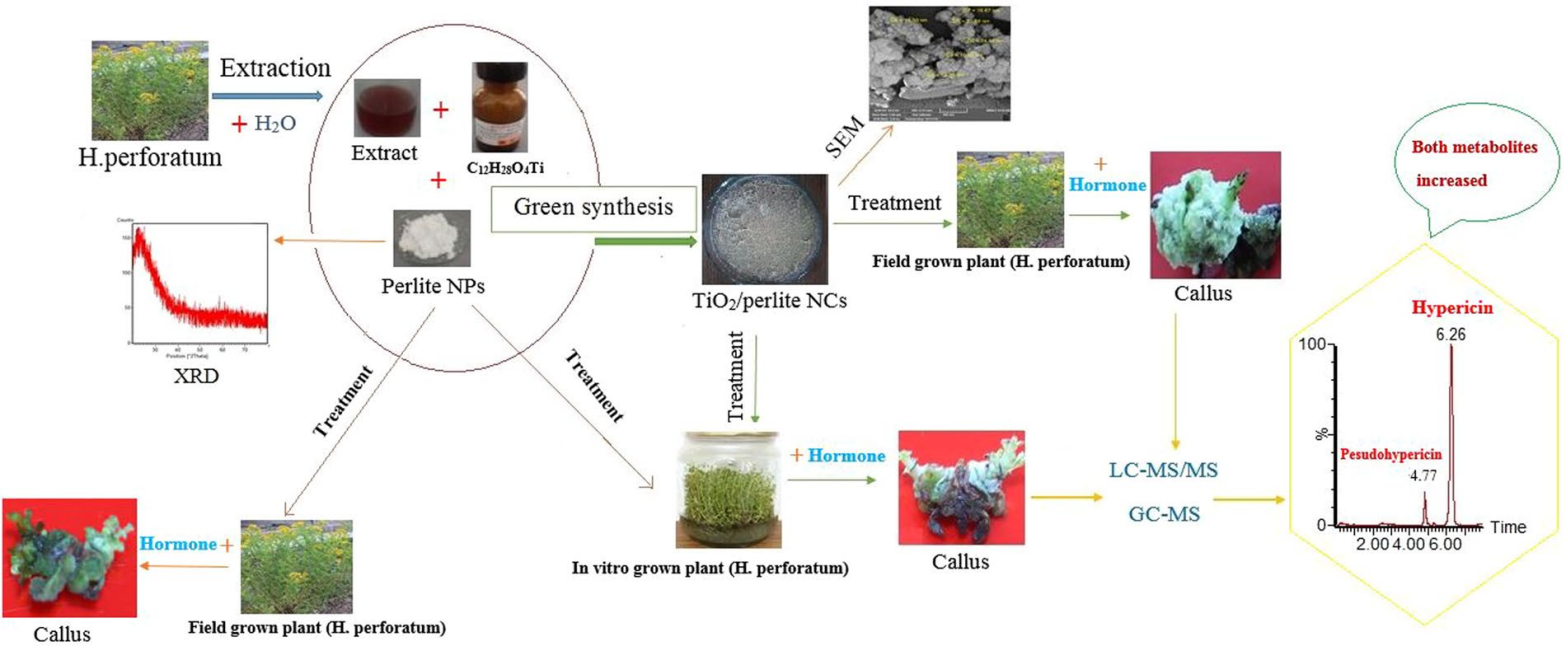
Schematic presentation of the different steps of the experimental work.

For induction of callus, nodal stem explants of *H. perforatum* were used. The fresh stem explants of field grown plants, collected from the herbarium of Tabriz University of Medical Sciences (East Azarbaijan, Tabriz, Iran), were sterilized by 70% ethanol for 1 min and 20% sodium hypochlorite solution for 15 min, followed by washing with sterile deionized water. In order to obtain *H. perforatum* seedlings, the surface of the seeds was also sterilized using the method mentioned above. Then the sterilized seeds were incubated on Murashige and Skoog (MS) medium (3% sucrose, 0.8% agar, pH 5.6–5.8). 24-day-old seedlings were used as the source of the explant. In order to induce callus formation, the obtained explants from both *in vitro* and field grown plants were cut into approximately 1 cm segments and transferred on the solid MS medium containing 1 mg/L of 2,4-D, 1 mg/L of 6-benzyl adenine (BA), and different concentrations of perlite NPs and TiO_2_/perlite NCs (0, 25, 50, 100, 150 and 200 mg/L). Callus cultures were kept under 16 h light and 8 h dark photoperiod at 24 ± 1 °C. The different experimental steps are schematically presented in Fig. 1.

### Biomass measurement

For each treatment, 30-day-old calli were used to measure the final fresh weight (FW) and dry weight (DW, dried at 35 °C for 24 h). Moreover, the number of shoots regenerated on calli was measured.

### Chlorophyll and carotenoid contents

An amount of 20 mg of callus was homogenized with 2 mL of dimethyl sulfoxide solvent and centrifuged at 8000 rpm for 15 min. Then, the obtained supernatant was separated and analyzed for Chlorophyll a, Chlorophyll b and total carotenoids (C_x + c_) contents by using UV-Vis spectrophotometer at 480, 649 and 665 nm^31^.

$$\begin{array}{c}\begin{array}{l}{{\rm{C}}}_{{\rm{a}}}=12.19\,{{\rm{A}}}_{665}-3.45\,{{\rm{A}}}_{649}\\ {{\rm{C}}}_{{\rm{b}}}=21.99\,{{\rm{A}}}_{649}-5.32\,{{\rm{A}}}_{665}\end{array}\\ {{\rm{C}}}_{x+c}=(1000{{\rm{A}}}_{480}-2.14\,{{\rm{C}}}_{{\rm{a}}}-70.16{{\rm{C}}}_{{\rm{b}}})/220\end{array}$$

### GC-Mass analysis

The volatile compounds of treated and untreated calli (600 mg) were isolated by n-hexane solvent (2 ml *3) and subjected to GC-Mass analysis using a fused silica capillary column (Elite-I, 30 m, 0.25 mm, 0.25 µm, 100% dimethylpolysiloxane) and a mass spectrometer 6890 (NMass selective detector/Agilent). The injection volume was 1 µL and the samples were analyzed under electron ionization energy of 70 eV. High purity (99.999%) helium, at a flow rate of 1 mL/min, was used as the carrier gas. The injector and ion-source temperature were set as 150 and 280 °C, respectively. The oven temperature was initially 50 °C for 3 min, then gradually increased to 120 °C at 10 °C/min (3 min), 150 °C at 10 °C/min (3 min), 220 °C at 7 °C/min (3 min), 290 °C at 7 °C/min (5 min), and finally increased to 300 °C and kept there for 2 min^32^. The volatile components were identified according to the comparison of their mass spectra with the NIST standard reference database. Standard of n-alkanes containing n and n + 1 carbons (Sigma) was used to calculate the retention indices (RI) using a generalized equation^33^.

### LC-MASS/MASS analysis

The dried powder of *H. perforatum* calli (20 mg) was extracted using 1 mL of methanol in an ultrasonic bath for 60 min. The obtained extracts were collected by centrifugation (8,000 rpm, 10 min) and filtration through a 0.45-µm filter. Finally, the samples were stored at −20 °C in the dark^34^. The separation of the components of the extract was done using an Alliance separations module 2695 (Waters, Milford, MA, USA), including a quaternary solvent delivery system, degasser, autosampler, column heater combined with a Quattro Micro API Triple Quadrupole LC-MS/MS (Waters, Micromass, Manchester, UK), and a Gemini column (50 × 2.00 mm, 5 micron). Chromatographic elution was performed using triethylammonium acetate buffer (0.01 M) at pH 7.0 as mobile phase A and the mixture of methanol and acetonitrile (50:50, *v/v*) as phase B, at a flow rate of 1.5 ml/min. The injection volume was 10 µL and the column temperature was maintained at 40 °C. Separation started with 40% A and 60% B (0–2 min), a linear gradient was applied up to 95% B and 5% A (2–4 min) and held for 5 min in this condition. The initial conditions were held for 1.5 min as a re-equilibration step. The total run time was 10.5 min.

Mass analysis was performed in negative ion mode. The ESI negative source values were: capillary voltage, 3.5 kV; cone, 60 V; extractor, 2 V; RF lens, 0.3 V; source temperature, 110 °C; desolvation temperature, 360 °C; desolvation gas and cone gas (nitrogen 99.99%) flow rates, 600 and 50 L/h, respectively. The analyzer settings were: resolution, 24.0 and 14.0 (unit resolution) for LM1 and LM2 resolution, respectively; 14.0 and 14.0 for HM1 and HM2 resolution, respectively; ion energy 0.5 and 1, respectively; entrance and exit energies, 60 and 60 (V); multiplier, 450 (V); collision gas (argon, 99.995%) pressure 1 × 10^−4^ mbar. The quantification of hypericin and pseudohypericin was performed by preparing different calibration standard solutions and recording the calibration curve. Hypericin and pseudohypericin (1 mg, Sigma) were dissolved in methanol (2 ml) and used as standard solution^27^. MassLynx 4.1 software was used to quantify the analyses result^35^. Since the analysis was performed with only one replicate, the data were qualitatively reported.

### Data analysis

All trials were directed under a completely randomized design by means of three replicates with 10 explants per treatment. Statistical analysis was carried out by using a one-way analysis of variance (ANOVA) based on Duncan’s comparison mean test (SPAS16, P ≤ 0.05).

## Results and Discussion

### Structural characterization of perlite NPs and TiO_2_/perlite NCs

**Figure 2:**
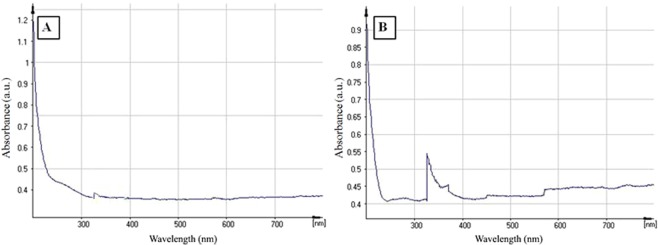
UV-Vis absorption spectra of perlite NPs (**A**) and TiO_2_/perlite NCs (**B**).

Structural features of perlite NPs and TiO_2_/perlite NCs were analyzed using UV-Vis spectroscopy, XRD, FESEM, EDX, TEM and FTIR techniques. UV–vis spectroscopy is an indirect technique to evaluate the fabrication of TiO_2_/perlite NCs from perlite NPs. Earlier reports stated that the maximum absorbance between 300–350 nm arises from the presence of green synthesized TiO_2_ NPs^36^ (Fig. 2A,B). When TiO_2_ nanoparticles are immobilized on the surface of perlite, the absorption shows maxima at 339 nm. Therefore, it confirms the formation of TiO_2_/perlite NCs (Fig. 2B).

**Figure 3:**
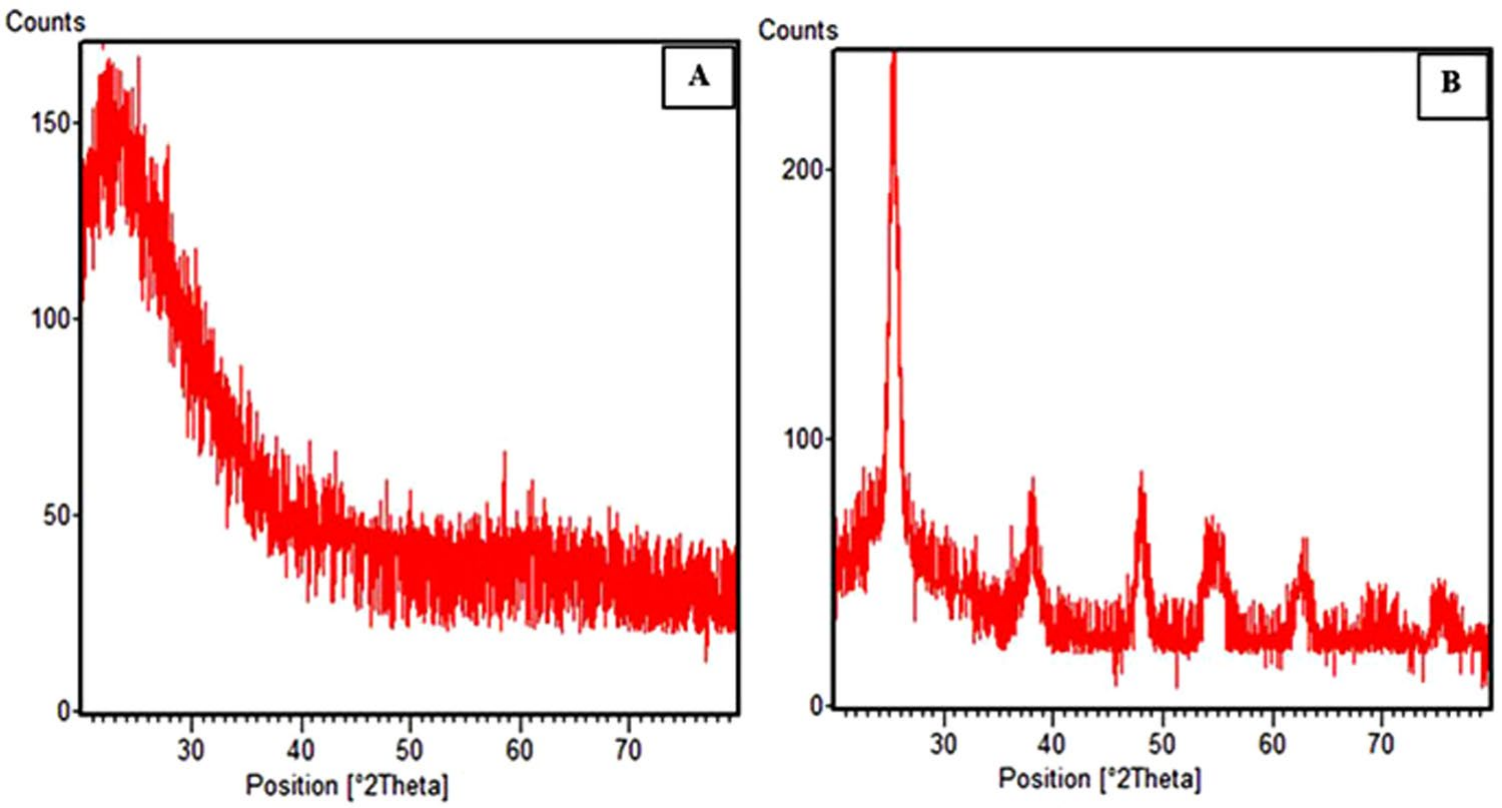
XRD patterns of perlite NPs (**A**) and TiO_2_/perlite NCs (**B**).

XRD pattern of perlite NPs indicated a characteristic peak at 2θ = 25° with an amorphous nature (Fig. 3A)^22^. The additional reflections at 2θ = 25.39° (101), 38.11° (004), 48.00° (200), 54.09° (105), 68.24 (116), 70.20° (220) were observed in the XRD pattern of TiO_2_/perlite NCs when TiO_2_ NPs were immobilized on the surface of perlite, which confirmed the anatase crystallite structure of TiO_2_ NPs and the tetragonal structure of TiO_2_/perlite NCs (Fig. 3B)^37^. All peaks in the diffractogram (Fig. 3B) were in good agreement with the standard spectrum (JCPDS no.: 88–1175 and 84–1286). The average crystallite size of perlite NPs and TiO_2_/perlite NCs were measured by Debye–Scherrer formula as 13.72 and 18.65 nm, respectively.

**Figure 4:**
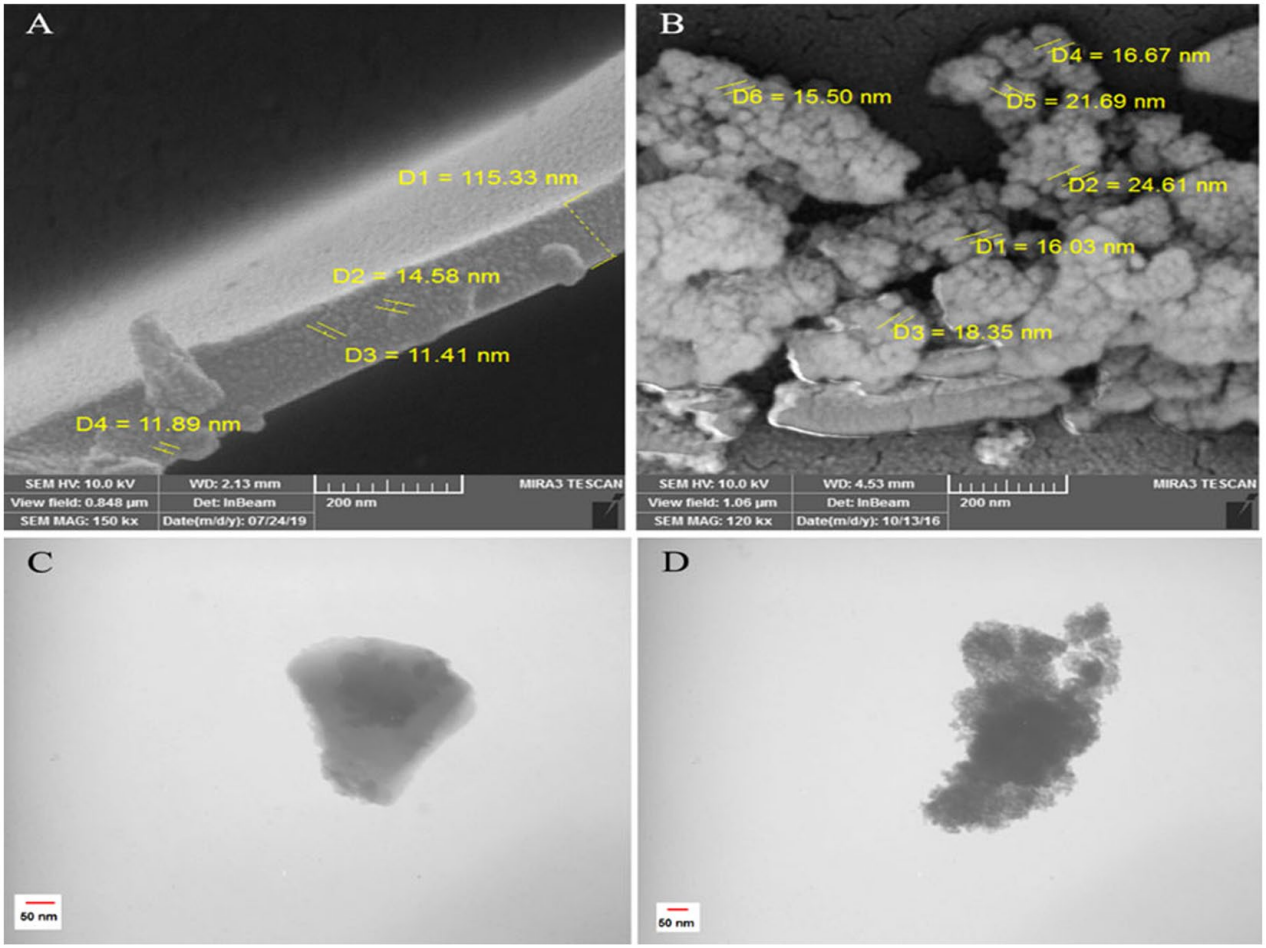
SEM images of perlite NPs (**A**) and TiO_2_/perlite NCs (**B**). TEM images of perlite NPs (**C**) and TiO_2_/perlite NCs (**D**).

The SEM and TEM images of as-synthesized perlite NPs and TiO_2_/perlite NCs are showed in Fig. 4A–D. These images (Fig. 4A,C) show the plate-shape and mesoporous entity for perlite NPs. In addition, as can be seen in Fig. 4D the perlite plates are entirely covered by TiO_2_ nanoparticles that appear as an aggregation of small spherical particles. Based on SEM image the morphology of as-synthesized TiO_2_ particles on perlite plate were with sizes ranging about 15.50–24.61 nm (Fig. 4B, Table 1).

**Table 1:** Colloidal properties of perlite NPs and TiO_2_/perlite NCs.

Sample	Size (nm)			PDI (μ_2_/Ƭ^2^)	Ζ (mV)
	XRD	SEM	DLS		
Perlite NPs	13.72	11.41–14.58	85.04–93.76	0.454	+30.09
TiO_2_/perlite NCs	18.65	15.50–24.61	168–173.4	0.576	+37.0

Size of the nanomaterials was measured based on XRD (calculated using Scherrer’s equation), SEM, and DLS (hydrodynamic diameter). Polydispersity index (PDI) and Zeta potential (Z) were determined based on DLS.

**Figure 5:**
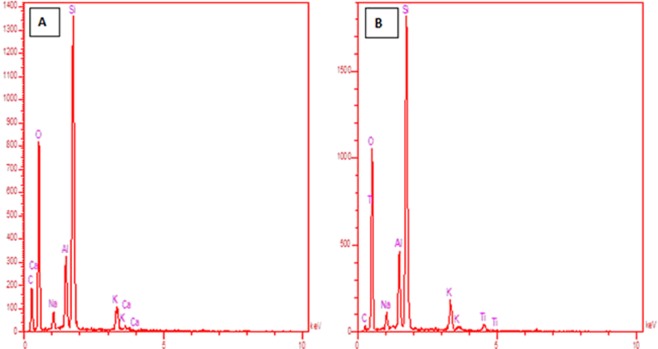
EDX spectrum of perlite NPs (**A**) and TiO_2_/perlite NCs (**B**).

According to the EDX spectrum of synthesized perlite NPs (Fig. 5A), it can be concluded that the silicon and aluminium were as the major elements because a higher amount of Si and Al are present in the profile. The presence of both Ti and O elements of the TiO_2_/perlite NCs was evident (Fig. 5B). The peaks of Ti seen on 0.6, 4.7 and 4.9 Kev. It appears that the presence of non-crystalline phytochemical substances which capped the TiO_2_/perlite NCs reduced the Ti:Si ratio (please see the discausion about FT-IR spectra and possible mechanism for the synthesis of TiO_2_/perlite NCs). Similar results for EDX patterns have been referred in literatures^38^. No other impurities were observed in EDX profile.

**Figure 6:**
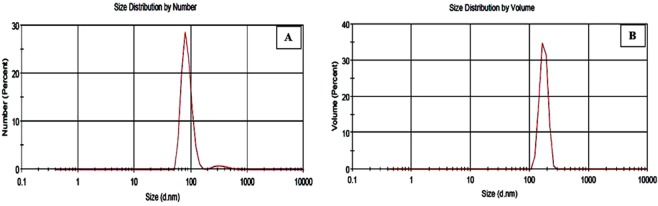
Size distribution graphs of perlite NPs (**A**) and TiO_2_/perlite NCs (**B**) based on DLS analysis.

According to the DLS analysis, the size distribution and zeta potential of nanomaterials were 85.04–93.76 nm and +30.09 mV for perlite NPs and 168–173.4 nm and +37 mV for TiO_2_/perlite NCs, respectively (Fig. 6, Table 1). The size measurements using DLS are basically determined by the hydrodynamic diameter of NPs, which depends not only on the core of the NP but also on surface coating and ion concentration in the medium. For that reason, the particle size can be larger than the sizes obtained using the SEM and XRD. Consistent with the DLVO theory, the high repulsive force between the nanostructures, due to the high surface charge, inhibits their agglomeration. Therefore, the high values of zeta potential confirm the high dispersity and stability of the synthesized nanomaterials in the suspension.

**Figure 7:**
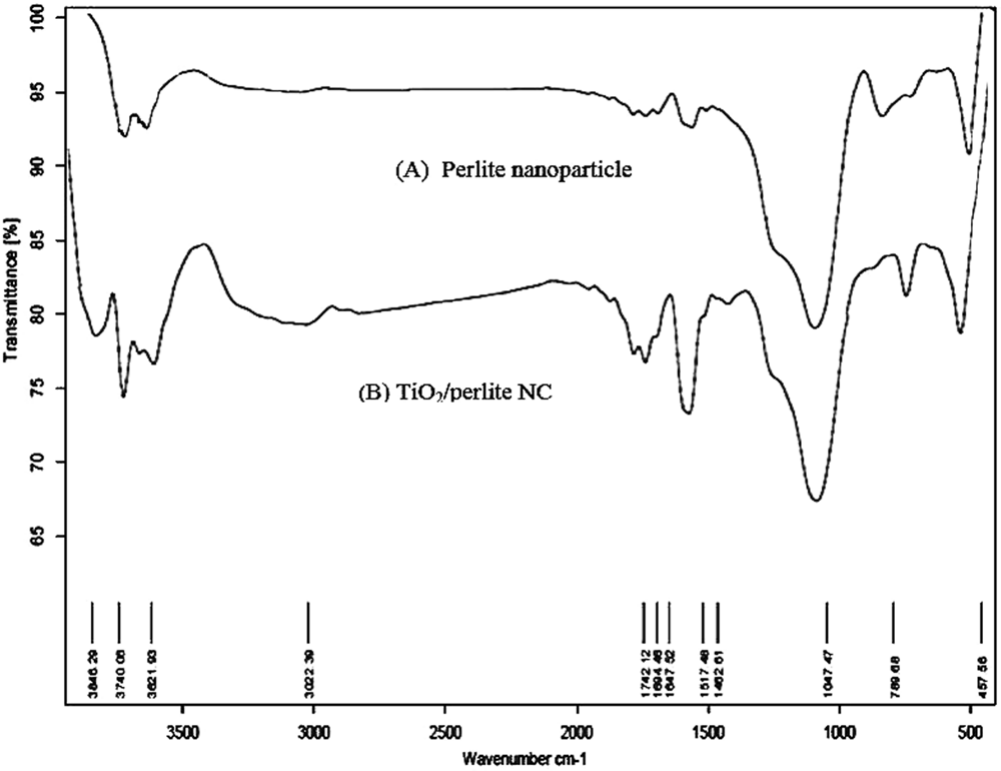
FT-IR patterns of perlite NPs (**A**) and TiO_2_/perlite NCs (**B**).

The FT-IR spectra of perlite NPs and TiO_2_/perlite NCs can be seen in Fig. 7. At FT-IR spectra of perlite NPs: The bonds at 457 and 1047 cm^−1^ corresponded to Al-O and Si-O stretching vibration bond, respectively. These peaks are the main features in perlite and other aluminosilicate phases^39^. The bands 3621 and 3740 cm^−1^ can also result from water bound directly to Si-O-H and Al-O-H (strongly bound to a surface as inner sphere complexe^39^ (Fig. 7A). At FT-IR spectra of TiO_2_/perlite NCs: the FTIR spectrum of TiO_2_ NPs clearly shows tree bands. The first band is the peak on 789 cm^−1^ was assigned to the Ti-O stretching bands. The second band is observed around 1610 and 1742 cm^−1^, corresponding to C=C and C=O of the aromatic ring and carbonyl functional groups, respectively. These bonds can be resulted from the functional groups of secondary metabolites of the extract^22,29^. It appears that the secondary metabolites of the extract, such as hyperforin, containing C=C and C=O groups, could be as a capping and stabilizing agent. It is due to the fact that the C=C and C=O groups of these secondary metabolites have a strong affinity to bind metals then can act as encapsulating agent and accordingly prevent the agglomeration of TiO_2_/perlite NCs^29^. The third is prominent peaks at 17423621, 3740 and 3846 cm^−1^ related to water bound directly to Si-O-H, Al-O-H and Ti-O-H, respectively^39^ (Fig. 7B).

### The possible mechanism for the synthesis of TiO_2_/perlite NCs

**Figure 8:**
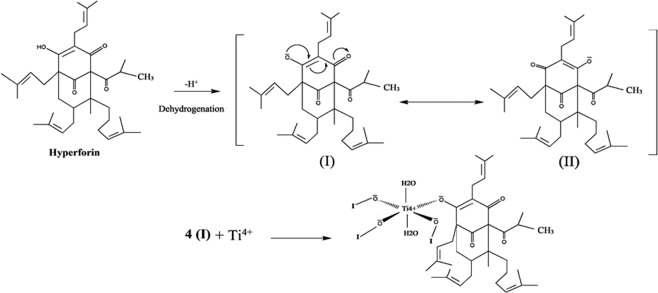
Possible mechanism for the synthesis of TiO_2_/perlite NC.

According to the available data about the biosynthesis of metal oxide NPs by using different plant extracts, a precise mechanism for the synthesis of NPs has not yet been approved. However, polar groups seem to be possible candidates for the biosynthesis of these NPs^29,40,41^. Hyperforin is the major of the two acylphloroglucinols that present in *H. perforatum*^42^. The adapted mechanism related to the capping effect of the plant extract is depicted in Fig. 8. Apparently, the vacant orbital of Ti^4+^ can be occupied by the lone pair electrons of the polar groups of molecule I. Afterwards, capping of Ti^4+^ ion by polar groups of the plant extract organizes a complex composite formation. Finally, calcination led to the synthesis of TiO_2_/perlite NCs in the reaction.

### The effect of perlite NPs and TiO_2_/perlite NCs on growth parameters

**Figure 9:**
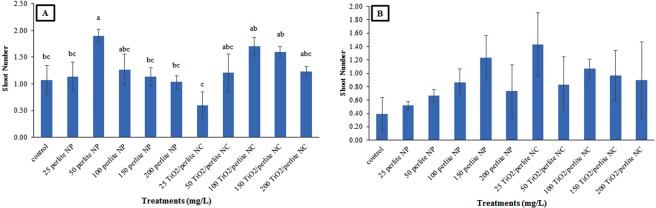
The effect of perlite NPs and TiO_2_/perlite NCs on fresh weight of calli obtained from *in vitro* grown (**A**) and field grown plants (**B**). Different letters indicate significant differences at p ≤ 0.05. The error bars represent standard error of the mean.

In order to investigate the effects induced by the synthesized nanomaterials on callus growth, we exposed the callus cultures of *H. perforatum* to different concentrations of perlite NPs and TiO_2_/perlite NCs. Total biomass of the callus cultures (fresh weight) was measured after 30 days of culture with and without the nanomaterials. In the cultures obtained from *in vitro* grown plants, as presented in Fig. 9A, both perlite NPs and TiO_2_/perlite NCs affected *H. perforatum* callus growth. Perlite NPs at the concentration of 50 mg/L and TiO_2_/perlite NCs at the concentrations of 100 and 200 mg/L significantly enhanced the callus growth by 104%, 108%, and 52% when compared to the control, respectively. In contrast, the callus cultures obtained from field grown plants showed no significant difference in fresh biomass of treated cultures, compared to the control (Fig. 9B). However, compared to the all treated cultures, highest biomass was observed in the calli under 100 mg/L of TiO_2_/perlite NCs.

**Figure 10:**
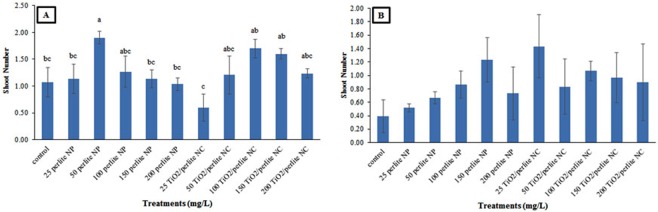
The effect of perlite NPs and TiO_2_/perlite NCs on shoot number of the calli obtained from *in vitro* grown (**A**) and field grown (**B**) plants. Different letters indicate significant differences at *p* ≤ 0.05. The error bars represent standard error of the mean. Figure 10B shows statistically non-significant results (*p* > 0.05).

**Figure 11:**
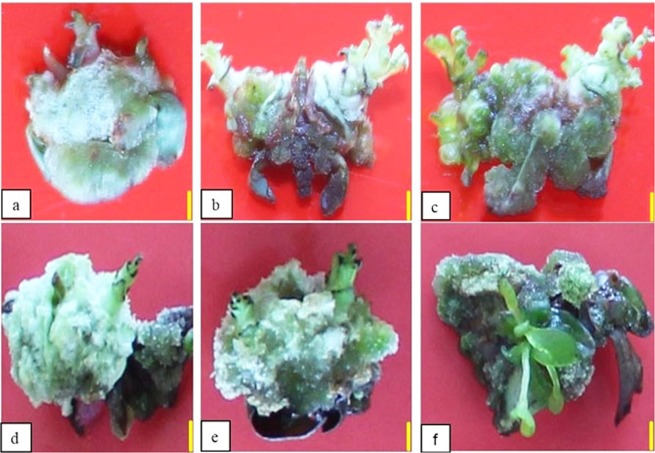
Calli obtained from *in vitro* grown plants: control (**a**), callus treated with 50 mg/L of perlite NPs (**b**), callus treated with 25 mg/L of TiO_2_/perlite NCs (**c**). Calli obtained from field grown plants: control (**d**), callus treated with 50 mg/L of perlite NPs (**e**), callus treated with 25 mg/L of TiO_2_/perlite NCs (**f**). Scale bar = 4 mm.

Regarding the number of shoots per callus in the cultures obtained from *in vitro* grown plants, only 25 mg/L of TiO_2_/perlite NCs showed the lowest shoot number in comparison to the control callus cultures. Nevertheless, in cultures obtained from field grown plants, both perlite NPs and TiO_2_/perlite NCs had no effect on the shoot number (Figs 10, 11).

According to the available data, different plant species show various responses to TiO_2_ NPs regarding their growth parameters^43^. Consistent with our results, some studies have indicated that TiO_2_ NPs have positive effects on plants growth. For instance, the results reported by Dehkourdi and Mosavi^44^ showed that nano-anatase (TiO_2_) caused a significant increase in the seed germination and biomass of *Petroselinum crispum* seedlings. It has also been reported that TiO_2_ NPs promote the growth at a suitable concentration in wheat seedlings grown in soil^45^. Nanoperlite at the concentration of 150 mg/L increased the shoot number in *Melissa officinalis* plant organ cultures which was attributed to the beneficial properties of perlite such as improvement of nutrient uptake and aeration in the culture medium^22^.

### The effects of perlite NPs and TiO_2_/perlite NCs on photosynthetic pigments content

**Figure 12:**
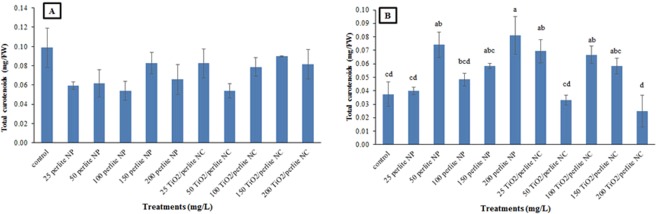
The effect of perlite NPs and TiO_2_/perlite NCs on the total carotenoids (C_x + c_) contents of calli obtained from *in vitro* grown (**A**) and field grown (**B**) plants. Different letters indicate significant differences at *p* ≤ 0.05. The error bars represent standard error of the mean. Figure 12A shows statistically non-significant results (*p* > 0.05).

The contents of chlorophyll a, chlorophyll b, and total carotenoids (C_x + c_) of *H. perforatum* calli were measured after treatment with different concentrations of perlite NPs and TiO_2_/perlite NCs. According to our results, applied nanomaterials had no effect on photosynthetic pigments content of *in vitro* grown calli (Fig. 12A). Related to the cultures obtained from field grown plants, there were no statistically significant difference in chlorophyll a and chlorophyll b contents between the untreated calli and those treated with perlite NPs and TiO_2_/perlite NCs. However, total carotenoids total carotenoids (C_x + c_) content increased in calli after exposure to 200 mg/L of perlite NPs, as well as 25 and 100 mg/L of TiO_2_/perlite NCs (Fig. 12B). An increase in the contents of photosynthetic pigments has been reported in plants treated with TiO_2_ NPs. TiO_2_ NPs promoted chlorophyll formation, photosynthetic rate, and growth in spinach plants^46^. Increased photosynthetic rate by TiO_2_ NPs has also been reported in *Vigna unguiculata* plant^26^.

### The effects of perlite NPs and TiO_2_/perlite NCs on volatile compounds

The variability in the composition of the volatile compounds of *H. perforatum* calli cultures was examined after exposure to perlite NPs and TiO_2_/perlite NCs. The GC-MS analysis showed 50 and 31 compounds in the extract of the calli obtained from *in vitro* grown and field grown plants, respectively (Tables 2, 3). Our results revealed the existence of hydrocarbons (aliphatic, aromatic and operating agent), alkaloids, phenolics, fatty acids, steroids and terpenes in the callus extracts. Alkaloids (such as 1,4-Phthalazinedione and 2,3-dihydro-6-nitro) were the main volatile constituents of the calli obtained from *in vitro* grown and field grown plants (Tables 2, 3). For calli obtained from *in vitro* grown plants, the control and treatment with 150 mg/L of TiO_2_/perlite NCs with 5 compounds and the treatment of 100 mg/L of perlite NPs with 20 compounds had the lowest and highest number of volatile compounds, respectively. The volatile compounds of the calli treated with 100 mg/L of perlite NPs contained a high number of aliphatic hydrocarbons (17 types of hydrocarbons). Only the callus cultures of control produced steroid (stigmasta-3,5-diene, 33.9%). A high percentage of alkaloids (84.43%) was determined in the treatment of 25 mg/L perlite NPs. Among the all treatments, 100 and 200 mg/L of perlite NPs significantly increased fatty acids by 4.41% and 12.19%, respectively, but control calli did not have these fatty acids. Only the cultures treated with 100 mg/L of perlite NPs produced sesquiterpene (1.9%) and diterpene (7.2%). Generally, the variety of volatile compounds in both perlite NPs and TiO_2_/perlite NCs treatments was higher than the control. However, the variety of compounds in perlite NPs treatments was higher than the TiO_2_/perlite NCs treatments.

**Table 2:** Chemical composition of the extract of *H. perforatum* calli obtained from *in vitro* grown plants after treatment with perlite NPs and TiO_2_/perlite NCs.

>Compunds	Peak area %											Classification	RT	RI
	Control	P 25 (mg/L)	P 50 (mg/L)	P 100 (mg/L)	P 150 (mg/L)	P 200 (mg/L)	P + T 25 (mg/L)	P + T 50 (mg/L)	P + T 100 (mg/L)	P + T 150 (mg/L)	P + T 200 (mg/L)			
Decane	—	—	—	2.16	—	—	—	—	—	—	—	Hydrocarbon-alkan	7.28	962
4,7-Methano-1H-indene, 3a,4,7,7a-tetrahydro-	—	—	—	2.09	—	—	—	—	—	—	—	Hydrocarbon	7.88	994
Undecane	—	—	—	1.57	—	—	—	—	—	—	—	Hydrocarbon-alkan	9.31	1075
1. 3-Cyclohexene-1-acetaldehyde	3.39	—	—	—	—	—	—	—	—	—	—	Aldehyde	9.32	1076
(1RS,2RS,4RS)−2-Methylbicyclo[2.2.2]oct-5-en-2-ol	—	—	—	1.09	—	—	—	—	—	—	—	Alcohol	9.39	1077
Dodecane	—	—	—	3.21	—	—	—	—	—	—	—	Hydrocarbon-alkan	11.2	1180
Tridecane	—	—	—	4.53	4.45	—	—	—	—	—	—	Hydrocarbon-alkan	13	1258
Tetradecane	—	—	—	—	—	6.48	—	—	—	—	—	Hydrocarbon-alkan	15.3	1360
Tridecane, 3-methyl-	—	—	—	1	—	—	—	—	—	—	—	Hydrocarbon-alkan	15.31	1361
Farnesan	—	—	—	1.9	—	—	—	—	—	—	—	Sesquiterpen	15.43	1367
Pentadecane	—	—	—	7.55	—	12.24	—	—	—	—	—	Hydrocarbon-alkan	17.91	1479
Hexadecane	—	—	—	8.67	—	12.26	—	—	—	—	—	Hydrocarbon-alkan	20.6	1584
Heptadecane	—	—	—	8.11	—	14	—	—	—	—	—	Hydrocarbon-alkan	23	1689
Heptadecane, 3-methyl	—	—	—	1.06	—	—	—	—	—	—	—	Hydrocarbon-alkan	24.39	1761
Carbamodithioic acid, dibutyl	—	—	—	—	4.2	—	—	—	—	—	—	Acid	24.43	1763
Octadecane	—	—	—	7.82	—	10.77	—	—	—	—	—	Hydrocarbon	24.9	1788
Hexadecane, 2,6,10,14-tetramethy	—	—	—	7.2	—	—	—	—	—	—	—	Diterpen	25.1	1798
Nonadecane	—	—	—	7.38	—	10.73	—	—	—	—	—	Hydrocarbon-alkan	26.6	1887
Hexadecanoic acid, methyl ester	—	—	—	4.41	—	4.99	—	—	—	—	—	Fatty acid	27.2	1925
3,11-diheptyloxybenzo[c]benzo[a]phenanthrene	—	—	10.48	—	—	—	4.12	—	—	—	—	phenolic	27.8	1963
Eicosane	—	—	—	7.02	—	7.3	—	—	—	—	—	Hydrocarbon-alkan	28.22	1991
Heneicosane	—	—	—	7.52	—	6.04	—	—	—	—	—	Hydrocarbon-alkan	29.7	2090
9-Octadecenoic acid, (E).Oleic acid	—	—	—	—	—	7.2	—	—	—	—	—	Fatty acid	29.82	2098
6-Aza-5,7,12,14-tetrathiapentacene	3.39	—	—	—	—	—	—	—	—	—	—	Alkaloid	30.22	2121
Docosane	—	—	—	3.52	—	—	—	—	—	—	—	Hydrocarbon-alkan	31.44	2190
Tricosane	—	—	—	2.3	—	—	—	—	—	—	—	Hydrocarbon-alkan	33.41	2292
Octadecane, 3-ethyl-5-(2-ethylbutyl)-	—	—	—	—	—	—	—	—	—	—	1.43	Hydrocarbon-alkan	35.2	2396
1,3,6,9b-Tetraazaphenalene-4-carbonitrile, 7,9-dibromo-2-(dibromomethyl)-	—	—	—	—	—	—	—	—	—	—	3.72	Alkaloid	36.38	2474
Pentacosane	—	—	—	—	—	—	—	—	—	7.91	—	Hydrocarbon-alkan	36.8	2502
1H-Indole, 2-methyl-3-phenyl-	—	—	—	—	—	—	—	—	—	—	2.58	Alkaloid-Indole	37.24	2534
N-METHYLDEACETYLCOLCHICINE	—	—	0.95	—	—	—	—	—	—	—	—	Alkaloid	37.47	2551
(Z,Z)-4-Ethyl-3-methyl-5-(5-4-aminophenyl-2-methylen)-3,4-dimethyl-5H-pyrrolyl-2-methylene)-3-pyrrolin-2-on	—	—	—	—	—	—	4.94	—	—	—	—	Alkaloid	37.7	2568
2-[4-Cyclohexylbutanoylamino]-3-chloro-1,4-naphthoquinone	—	1.48	—	—	—	—	—	3.17	—	—	—	Phenolic. Quinone	39.1	2678
phenaleno[2,3-g]quinolin-7-one	—	—	—	—	—	—	2.88	7.94	—	—	—	Alkaloid	39.4	2702
2,2,3,3-TETRAFLUORO-5-(1,1,2,2-TETRAFLUOROETHOXY)-2,3-DIHYDROBENZOFURANOBENZOFURAN	—	9.63	8.57	—	—	—	3.29	5.29	1.83	10.17	34.67	Phenolic	39.41	2703
Antra-9,10-quinone, 1-(3-hydrohy-3-phenyl-1-triazenyl)-	—	—	2.86	—	—	—	—	—	—	—	—	Phenolic. Quinone	39.43	2704
6H-phenanthro[9,8-gh]quinolin-6-one	—	2.96	—	—	—	—	—	20.11	—	—	—	Alkaloid	39.7	2728
2-Ethylacridine	—	—	2.86	—	—	—	—	—	—	—	—	Alkaloid	39.82	2739
5-Methyl-2-phenylindolizine	—	13.33	—	—	—	—	—	—	—	—	—	Alkaloid	40.4	2789
Cyclohexane-1,3-dione, 2-allylaminomethylene-5,5-dimethyl	—	—	—	—	5.19	—	—	—	—	—	—	Keton	40.5	2797
4-Methoxy-3-(3-methoxyphenyl)-4-methylpentan-1-ol	—	—	—	—	—	—	—	—	21.95	—	—	Alcohol	40.8	—
1,4-Phthalazinedione, 2,3-dihydro-6-nitro-	2.54	54.81	19.05	—	—	—	32.1	11.11	28.05	11.86	19.77	Luminole. Alkaloid	41.37	—
demethoxy-12-epi-fumitremorgin B	—	9.63	—	—	—	—	33.98	—	—	8.76	—	Alkaloid	41.4	—
7H-Dibenzo[b,g]carbazole, 7-methyl-	—	—	—	—	—	—	—	19.58	—	—	—	Alkaloid	41.45	—
1H-Indole-2-carboxylic acid, 6-(4-ethoxyphenyl)-3-methyl-4-oxo-4,5,6,7-tetrahydro-, isopropyl ester xo-4,5,6,7-tetrahydro-, isopropyl ester	—	—	52.38	—	—	—	—	—	—	—	—	Alkaloid-indol	41.47	—
Benzo[h]quinoline, 2,4-dimethyl-	—	3.7	—	—	3.09	—	—	6.88	35.37	—	—	Alkaloid	41.6	—
2-(Acetoxymethyl)-3-(methoxycarbonyl)biphenylene	—	—	—	—	37.68	—	—	—	8.54	—	27.51	Aromatic	43	—
Cyclopentanecarboxamide, 3-ethenyl-2-(3-pentenylidene)-N-phenyl-, [1.alpha.,2Z(E),3.alpha.]-	41.53	—	—	—	20.44	—	—	17.46	2.44	27.4	—	Aromatic ester	43.55	—
Stigmastan-3,5-diene	33.9	—	—	—	—	—	—	—	—	—	—	Steroid	44.3	—
Total identification	84.75	95.54	97.15	95.75	75.45	92.01	80.6	95.77	98.18	82.74	96.85			
Fatty acids	—	—	—	4.41	—	12.19	—	—	—	—	—			
Hydrocarbons-aliphatic	—	—	—	70.51	4.45	79.82	—	—	—	7.91	5.73			
Hydrocarbons-aromatic	41.53	—	—	—	58.12	—	—	17.46	10.98	27.4	27.51			
Operating agant hydrocarbon	3.39	—	—	1.09	5.59	—	—	—	21.95	—	—			
Alkaloids	5.93	84.43	75.24	—	3.09	—	73.19	69.85	63.42	20.62	28.94			
Phenolic compounds	—	11.11	21.91	—	—	—	7.41	8.46	1.83	26.81	34.67			
Sesquiterpenes	—	—	—	1.9	—	—	—	—	—	—	—			
Steroids	33.9	—	—	—	—	—	—	—	—	—	—			
Carboxylic acid	—	—	—	—	4.2	—	—	—	—	—	—			
Diterpenes	—	—	—	7.2	—	—	—	—	—	—	—			

P: Perlite NPs. P + T: TiO_2_/perlite NCs.

**Table 3:** Chemical composition of the extract of *H. perforatum* calli obtained from field grown plants after treatment with perlite NPs and TiO_2_/perlite NCs.

Compunds	Peak area %											Classification	RT	RI
	Control	P 25 (mg/L)	P 50 (mg/L)	P 100 (mg/L)	P 150 (mg/L)	P 200 (mg/L)	P + T 25 (mg/L)	P + T 50 (mg/L)	P + T 100 (mg/L)	P + T 150 (mg/L)	P + T 200 (mg/L)			
Pentadecane	—	—	—	4.75	—	—	—	—	—	—	—	Hydrocarbon-alkan	17.91	1479
Hexadecane	—	—	—	7.31	—	—	—	—	—	—	—	Hydrocarbon-alkan	20.6	1584
2-Benzyl-1,2,3,4-tetrahydro-1-phenethylidene-.beta.-carboline	—	—	—	2.53	—	—	—	—	—	—	—	Alkaloid	20.86	1593
Heptadecane	—	—	—	8.04	—	—	—	—	—	—	—	Hydrocarbon—alkan	23	1689
Octadecane	—	—	—	10.54	—	—	—	—	—	—	—	Hydrocarbon—alkan	24.9	1788
Nonadecane	—	—	—	10.9	—	—	—	—	—	—	—	Hydrocarbon—alkan	26.6	1887
Hexadecanoic acid, methyl ester (CAS)	—	—	—	14.34	—	—	—	—	—	—	—	Fatty acid	27.2	1924
3,11—diheptyloxybenzo[c]benzo[a]phenanthrene	—	2.27	—	3.38	—	—	—	—	—	—	—	Phenolic	27.8	1963
Eicosane	—	—	—	11.57	—	—	—	—	—	—	—	Hydrocarbon—alkan	28.22	1991
7-Acetoxyeicosane	—	—	—	5.21	—	—	—	—	—	—	—	Ester	28.58	2015
Heneicosane	—	—	—	8.07	—	—	—	—	—	—	—	Hydrocarbon—alkan	29.7	2090
Octadecane, 3-ethyl-5-(2-ethylbutyl)-	0.5	2.27	—	—	—	—	—	—	—	—	—	Hydrocarbon	35.2	2296
Tetracosane	—	—	—	2.07	—	—	—	—	—	—	—	Hydrocarbon-alkan	35.28	2401
Hexadecane, 8-hexyl-8-pentyl	—	6.82	—	—		—	—	—	—	—	—	Hydrocarbon-alkan	35.64	2425
Pentacosane	—	—	—	3.35	—	—	—	—	—	—	—	Hydrocarbon-alkan	36.8	2501
1,2-Benzenedicarboxylic acid, ditridecyl ester	—	—	—	7.1	—	—	—	—	—	—	—	Acid	37.49	2553
(Z,Z)-4-Ethyl-3-methyl-5-(5-4-aminophenyl-2-methylen)-3,4-dimethyl-5H-pyrrolyl-2-methylene)-3-pyrrolin-2-on	—	—	—	—	—	3.8	—	—	—	—	—	Alkaloid	37.7	2568
phenaleno[3,2-f]quinolin-7-one	—	—	—	—	—	—	—	—	—	12.14	—	Alkaloid	39.21	2686
2,2,3,3-TETRAFLUORO-5-(1,1,2,2-TETRAFLUOROETHOXY)-2,3-DIHYDROBENZOFURANOBENZOFURAN	4.81	15.91	3.81	-	16.06	—	2.56	0.89	—	12.86	—	Phenolic	39.41	2703
6H-phenanthro[9,8-gh]quinolin-6-one	—	—	5.71	—	—	—	—	1.53	—	—	—	Alkaloid	39.7	2728
(S)-(E)-(-)-4-Acetoxy-1-phenyl-2-dodecen-1-one	—	—	—	—	—	2.38	—	—	—	—	—	Ester	40.19	2770
1,2-Benzenediol, 3,5-bis(1,1-dimethylethyl)-	—	—	—	—	—	—	—	—	—	—	8.82	Phenolic	40.24	2775
5-Methylthio-7,8-dihydro-6H-benzocyclohepta[2,1-e]pyrazolo[1,5-a]pyrimidine	9.45	—	—	—	15.03	—	—	—	—	—	—	Alkaloid	40.29	2779
Cyclohexane-1,3-dione, 2-allylaminomethylene-5,5-dimethyl	—	—	—	—	6.74	—	6.11	—	—	—	—	Hydrocarbon-alkan	40.5	2797
1,2,4-Benzenetricarboxylic acid, 4-dodecyl dimethyl ester	1.99	27.27	—	—	—	—	—	11.32	—	—	—	Carboxylic acid	40.59	—
4-Methoxy-3-(3-methoxyphenyl)-4-methylpentan-1-ol	—	—	—	—	—	—	—	2.29	—	—	—	Alcohol	40.8	—
1,4-Phthalazinedione, 2,3-dihydro-6-nitro	30.51	11.36	—	—	—	5.94	53.65	—	—	30.71	—	Alkaloid	41.37	—
Benzo[h]quinoline, 2,4-dimethyl-	3.48	—	—	—	—	—	4.73	—	2.6	—	—	Alkaloid	41.4	—
7H-Dibenzo[b,g]carbazole, 7-methyl	—	—	33.4	—	—	—	—	—	—	—	—	Alkaloid	41.5	—
6-methylthio[1]benzothieno[2,3-c]quinoline	—	—	37	—	—	—	—	—	—	—	—	Alkaloid	42.07	—
2-(Acetoxymethyl)-3-(methoxycarbonyl)biphenylene	15.26	—	—	—	—	52.97	26.23	52.29	79.69	—	—	Aromatic	43	—
Cyclopentanecarboxamide, 3-ethenyl-2-(3-pentenylidene)-N-phenyl-, [1.alpha.,2Z(E),3.alpha.]-	18.57	27.27	—	—	38	26.13	—	24.81	—	17.86	42.65	Aromatic	43.55	—
Total identification	84.57	70.88	79.92	92.06	89.82	91.22	93.29	83.21	82.29	73.57	51.47			
Fatty acids	—	—	—	14.34	—	—	—	—	—	—	—			
Hydrocarbons-aliphatic	0.5	13.64	—	66.6	3.8	—	—	1.4	—	—	—			
Hydrocarbons-aromatic	33.83	25	—	—	48.19	79.1	26.23	77.1	79.69	17.86	42.65			
Operating agant hydrocarbon	—	2.27	—	5.21	6.74	2.38	6.11	2.29	—	—	—			
Alkaloids	43.44	11.36	76.11	2.53	15.03	9.74	58.38	1.53	2.6	42.85	—			
Phenolic compounds	4.81	18.61	3.81	3.38	16.06	—	2.57	0.89	—	12.86	8.82			
sesquiterpenes	—	—	—	—	—	—	—	—	—	—	—			
Steroids	—	—	—	—	—	—	—	—	—	—	—			
Carboxylic acid	1.99	—	—	7.1	—	—	—	11.32	—	—	—			
diterpenes	—	—	—	—	—	—	—	—	—	—	—			

P: Perlite NPs. P + T: TiO_2_/perlite NCs’.

The GC-MS results of volatile compounds of the calli obtained from field grown plants (Table 3) indicated that alkaloid and aliphatic hydrocarbon compounds exist in all treatments. The highest number of compounds was observed in 100 mg/L of perlite NPs treatment (15 compounds) and the lowest number of compounds was observed in 200 mg/L of TiO_2_/perlite NCs treatment (2 compounds). Among the all perlite NPs and TiO_2_/perlite NCs treatments, only 100 mg/L of perlite NPs induced the production of fatty acid (hexadecanoic acid, methyl ester, 14.34%). Control and treatments with 100 mg/L of perlite NPs and 50 mg/L of TiO_2_/perlite NCs showed a significant increase in the production of carboxylic acids. The extracts of the calli treated with 50 mg/L of perlite NPs contained a large number of alkaloids (76.11%). All treatments except 200 mg/mL of TiO_2_/perlite NCs were able to produce alkaloids. Phenolic compounds were observed in all treatments except for 200 mg/L of TiO_2_/perlite NCs and 100 mg/L of perlite NPs. According to the GC-MS results, calli obtained from *in vitro* grown plants produced more volatile compounds relative to the calli obtained from field grown plants under the nanomaterial stress conditions. The stress caused by perlite NPs and TiO_2_/perlite NCs led to an increase in the variety, amount and number of volatile compounds in both calli.

In accordance with our results, some other studies have reported the potential of TiO_2_ NPs in the modulation of volatile compounds content of plants. Mohammad *et al*.^47^ demonstrated that foliar application of TiO_2_ NPs considerably augmented shoot dry mass and essential oil content of *Dracocephalum moldavica* L. under normal irrigation and water-deficit stress. The GC–MS revealed that *S. officinalis* plants enhanced plant dry matter and essential oils content after exposure to nano-TiO_2_^27^. Regarding the industrial application of essential oils, utilizing effective elicitors can be a beneficial approach to augment the production of useful secondary metabolites.

### The effects of perlite NPs and TiO_2_/perlite NCs on the production of hypericin and pseudohypericin

**Figure 13:**
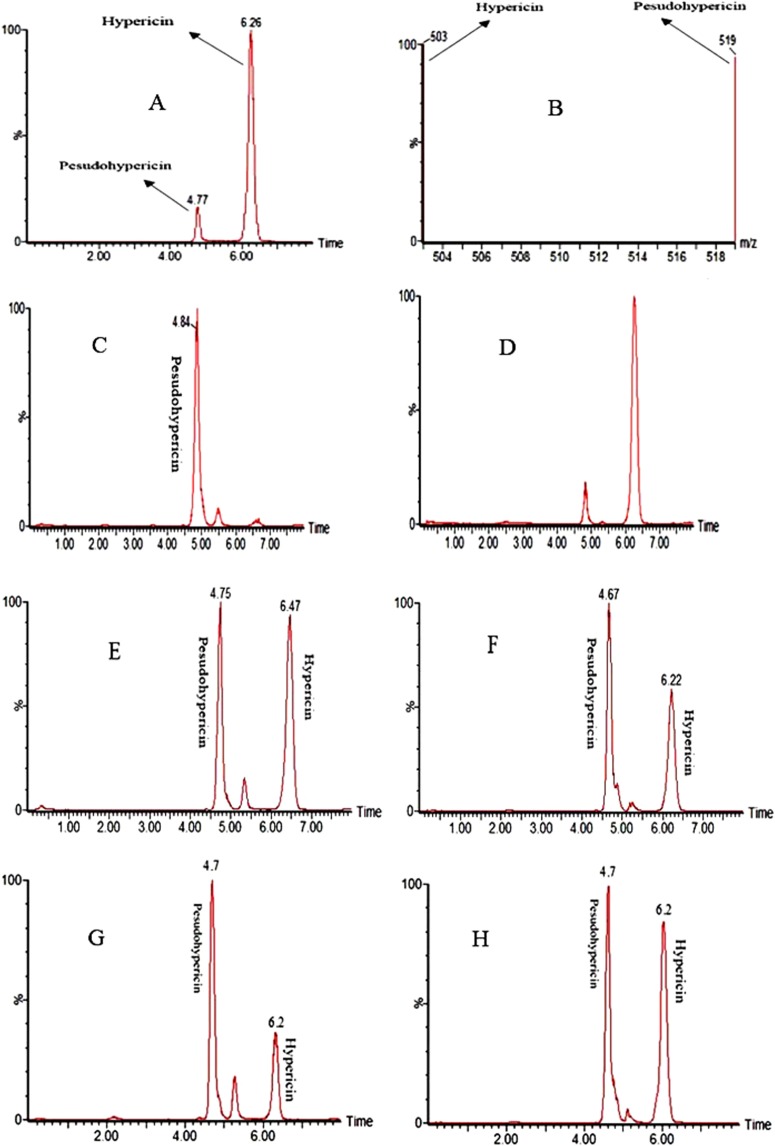
LC-MS chromatograms of *H. perforatum* callus extracts. Chromatogram (**A**) and mass spectrum (**B**) of hypericin and pseudohypericin standard solution. Chromatogram of the extracts of control calli obtained from *in vitro* grown (**C**) and field grown plants (**D**), calli obtained from *in vitro* grown plants treated with 100 mg/L of perlite NPs (**E**), calli obtained from field grown plants treated with 100 mg/L of perlite NPs (**F**), calli obtained from *in vitro* grown plants treated with 50 mg/L of TiO_2_/perlite NCs (**G**), and calli obtained from field grown plants treated with 50 mg/L of TiO_2_/perlite NCs (**H**).

The effects of perlite NPs and TiO_2_/perlite NCs on hypericin and pseudohypericin accumulation in callus cultures of *H. perforatum* were evaluated using LC-MS/MS. We compared the amount of desired compounds in the calli obtained from *in vitro* grown plants to those obtained from field grown plants. Retention time values (t_R_) for pseudohypericin and hypericin were 4.7 and 6.2 min, respectively (Fig. 13). The Values of m/z for pseudohypericin and hypericin were 519 and 503, respectively (Fig. 13). According to the results, only pseudohypericin was detected in the extract of control calli obtained from *in vitro* grown plants, although no hypericin and pseudohypericin were detected in the extract of control calli obtained from field grown plants. The production of hypericin by callus cultures initiated from *in vitro* grown *H. perforatum* was observed after treatment with 25 and 100 mg/L of perlite NPs. However, hypericin in callus cultures obtained from field grown *H. perforatum* was detected in 50, 100, and 200 mg/L of perlite NPs, as well as 25, 50, 150, and 200 mg/L of TiO_2_/perlite NCs. Pseudohypericin was also evident in the cultures obtained from *in vitro* grown plants treated with 25, 100, and 150 mg/L of perlite NPs and 50 and 100 mg/L of TiO_2_/perlite NCs. In the case of cultures initiated from field grown plants, pseudohypericin was observed in 50-200 mg/L perlite NPs and 25, 50, 150, and 200 mg/L TiO_2_/perlite NCs treatments (Fig. 13).

So far, some researches have been conducted to study the potential of chemical elicitors such as NPs on the manipulation of *H. perforatum* secondary metabolism. It has been revealed that chromium affected the production of protopseudohypericin, hypericin, and pseudohypericin in *H. perforatum* seedlings^48^. Supplementation of zinc and iron oxides NPs in *H. perforatum* cell cultures stimulated the production of hypericin and hyperforin^12^. In the same way, our results suggest that perlite NPs and TiO_2_/perlite NCs can possibly be considered as effective elicitors for the induction of hypericin and pseudohypericin production in callus cultures of *H. perforatum*.

## Conclusion

Our results indicated that employing nano-elicitors such as perlite NPs and TiO_2_/perlite NCs can stimulate the production and accumulation of secondary metabolites without having adverse impacts on the growth of *H. perforatum* callus cultures. Callus cultures obtained from *in vitro* grown plants supplemented with perlite NPs and TiO_2_/perlite NCs produced more volatile compounds than those obtained from field grown plants. Both perlite NPs and TiO_2_/perlite NCs were able to induce the production of hypericin and pseudohypericin in *H. perforatum* calli. Therefore, along with numerous well-known biotic and abiotic elicitors, the biosynthesized perlite NPs and TiO_2_/perlite NCs can be considered as a new class of elicitors. However, little is known about the induction of secondary metabolites in response to biosynthesized nanomaterials and more experimental data are required to provide insights to their application as elicitors.
